# Positive outcome of diaphragm covering and total pleural covering techniques for catamenial pneumothorax

**DOI:** 10.1093/jscr/rjad421

**Published:** 2023-07-20

**Authors:** Sachie Koike, Nobutaka Kobayashi, Masahisa Miyazawa

**Affiliations:** Department of Thoracic Surgery, Japanese Red Cross Society Nagano Hospital, Nagano, Nagano, Japan; Division of General Thoracic Surgery, Department of Surgery, Shinshu University School of Medicine, Matsumoto, Nagano, Japan; Department of Thoracic Surgery, Japanese Red Cross Society Nagano Hospital, Nagano, Nagano, Japan; Department of Thoracic Surgery, Japanese Red Cross Society Nagano Hospital, Nagano, Nagano, Japan

## Abstract

Catamenial pneumothorax (CP) is reported to be caused by the endometriosis of diaphragm, lung and parietal pleura. Therefore, the resection of endometriotic lesion in these organs is reported as effective surgical treatment. Overlooking of endometrial tissues during the operation is believed to be the cause of recurrence after surgical treatment. To address this problem, we underwent total diaphragm covering (TDC) and total pleural covering with sheets of oxidized regenerated cellulose mesh. This report described two CP cases that underwent total diaphragm covering (TDC) and total pleural covering. Both patients were followed up for 1 year without recurrence.

## INTRODUCTION

Catamenial pneumothorax (CP) is reported to be caused by the endometriosis of diaphragm, lung and parietal pleura. To prevent its recurrence, surgical resection of the endometrial tissues of these organs is performed as effective treatment [1]. However, the postsurgical recurrence rate is relatively high [1, 2], because of microscopic thoracic endometrial tissue overlooked during the operation, and the re-dissemination of endometrial tissue from pelvic endometriosis [1]. To prevent possible recurrence, we attempted to cover the entire surface of diaphragm and lung using total pleural covering (TPC) technique reported by Kurihara *et al*. [3] reported for lymphangioleiomyomatosis (LAM) management. Here we report two patients with CP who were successfully treated with total diaphragm covering (TDC) and TPC techniques.

## CASE REPORT

### Case 1

**Figure 1:**
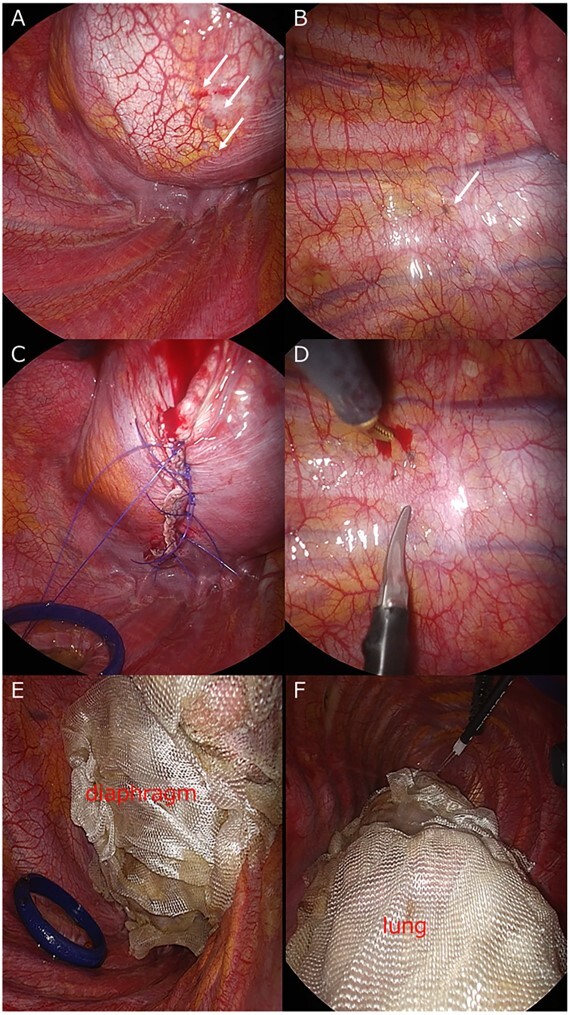
(**A**) Endometrial lesions of the diaphragm (arrow). (**B**) Endometrial lesions of the parietal pleura (arrow). (**C**) Diaphragm endometriosis is resected and hand sutures were added. (**D**) Lesions in the parietal pleura (arrow) are removed. (**E**) The entire surface of diaphragm and (**F**) the visceral pleura of lung is covered with ORC mesh.

A 42-year-old woman with a history of endometriosis who was treated with hormonal therapy visited our hospital because of recurring pneumothorax of the right lung. The last pneumothorax was 1 year ago. The pneumothorax persisted for 20 days with chest tube insertion, and video-assisted thoracoscopic surgery (VATS) was scheduled. During the surgery, endometrial lesions of the diaphragm and parietal pleura were observed (Fig. 1A and B). No lung lesions were observed. The diaphragm endometriosis was resected using an endoscopic stapler, and hand sutures were added (Fig. 1C). The lesions in the parietal pleura were removed by limited parietal pleurectomy with endoscopic scissors (Fig. 1D). The entire diaphragm was covered with oxidized regenerated cellulose (ORC) mesh sheets (TDC) (Fig. 1E). To prevent overlooking the microscopic thoracic endometrial tissues of the lung, we covered the entire lung using ORC mesh sheets (TPC) (Fig. 1F). The patient was followed up for 1 year without evidence of recurrence.

### Case 2

**Figure 2:**
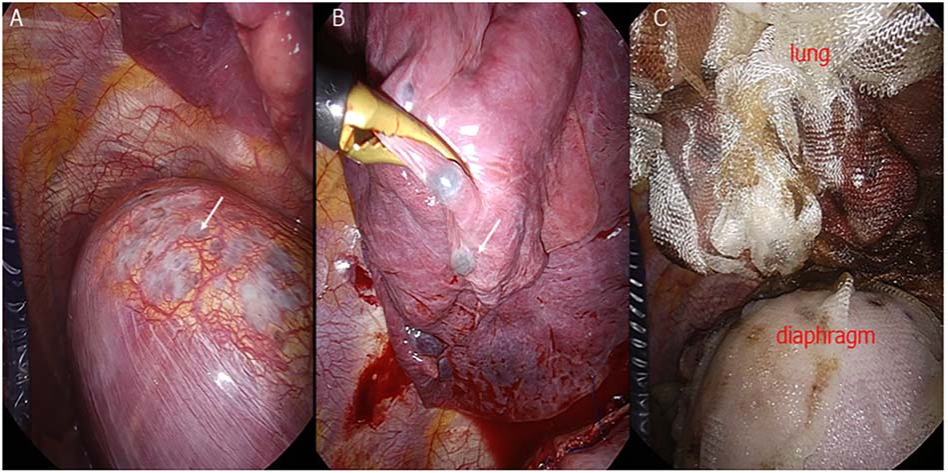
(**A**) Endometrial lesions of the diaphragm (arrow). (**B**) Endometrial lesions of the visceral pleura. (**C**) The entire diaphragm and lung are covered with ORC mesh.

Case 2 was a 40-year-old woman with a history of endometriosis treated with hormonal therapy who came to our hospital because of recurring pneumothorax. The last pneumothorax was 3 months ago. The pneumothorax persisted for 7 days with chest tube insertion, and VATS was scheduled. During the surgery, many endometrial lesions of the diaphragm and lung were observed (Fig. 2A and B). We resected as many lesions as possible using an endoscopic stapler, and TDC and TPC were performed to cover the residual lesions (Fig. 2C). The patient was followed up for 1 year without evidence of recurrence.

## COMMENTS

The recurrence rate after surgery of CP was relatively high (30–32%) [1, 2] compared with that of normal primary spontaneous pneumothorax (3–7%) [4] because of microscopic thoracic endometrial tissue overlooked during the operation, and the re-dissemination of endometrial tissue from pelvic endometriosis [1]. To prevent this, we attempted coverage using the TPC technique.

TPC is a surgical technique, which the surgeons cover the entire visceral pleura with ORC mesh for reinforcement, reported by Kurihara *et al* for the treatment of LAM. After TPC, the visceral pleura is approximately five times thicker than the untreated portions, whereas extensive pleural symphysis accompanying fibroblast proliferation and collagen deposition [3]. We hypothesized that this thickened pleura may cover the overlooked or re-disseminated endometrial tissues, and extended its application to the surface of the diaphragm.

In this report, both patients who were treated with TDC and TPC were followed up for 1 year without evidence of recurrence. This suggests that the effectiveness of the treatments. In Case 2, there were more endometrial lesions than we could resect, and the residual lesions were covered with ORC mesh. The positive outcome of Case 2 suggests that TDC and TPC method may also be effective for cases that endometrial lesions could not be completely resected.

In conclusion, TDC and TPC methods may be effective in reducing CP recurrence. Further treatment using this strategy may be performed and reported.

## CONFLICT OF INTEREST STATEMENT

None declared.

## FUNDING

None.

## DATA AVAILABILITY

The data underlying this article cannot be shared publicly or protecting privacy of individuals that participate in this study. The data may be shared on reasonable request to the corresponding author after additional approval by the Institutional Review Board of Japanese Red Cross Society Nagano Hospital.
